# Aluminum salts as an adjuvant for pre-pandemic influenza vaccines: a meta-analysis

**DOI:** 10.1038/s41598-018-29858-w

**Published:** 2018-07-30

**Authors:** Yu-Ju Lin, Yun-Jui Shih, Chang-Hsun Chen, Chi-Tai Fang

**Affiliations:** 1 Division of Preparedness and Emerging Infectious Diseases, Centers for Disease Control, Taipei, 100 Taiwan; 2 Institute of Epidemiology and Preventive Medicine, College of Public Health, National Taiwan University, Taipei, 100 Taiwan; 3 Division of Infectious Diseases, Department of Internal Medicine, National Taiwan University Hospital, Taipei, 100 Taiwan

## Abstract

Avian-origin H5/H7 influenza has the potential to cause the next influenza pandemic. Availability of effective vaccines is an essential part of pre-pandemic preparedness. However, avian influenza surface antigens are poorly immunogenic to humans, which necessitates the use of adjuvants to augment the immunogenicity of pre-pandemic influenza vaccines. Aluminum salts are approved, safe, and affordable adjuvants, but their adjuvanticity for influenza vaccines remains unverified. We conducted the first meta-analysis on this issue. A total of nine randomized controlled trials (2006–2013, 22 comparisons, 2,467 participants in total) compared aluminum-adjuvanted H5N1 vaccines versus non-adjuvanted counterparts. The weighted estimate for the ratio of the seroprotection rate after a single dose of H5N1 vaccine is 0.66 (95% CI: 0.53 to 0.83) by hemagglutination-inhibition assay or 0.56 (95% CI: 0.42 to 0.74) by neutralizing titer assay. The weighted estimate for the risk ratio of pain/tenderness at injection sites is 1.85 (95% CI: 1.56 to 2.19). The quality of evidence is low to very low for seroprotection (due to indirectness and potential reporting bias) and moderate for pain/tenderness (due to potential reporting bias), respectively. The significantly lower seroprotection rate after aluminum-adjuvanted H5N1 vaccines and the significantly higher risk of pain at injection sites indicate that aluminum salts decrease immunogenicity but increase local reactogenicity of pre-pandemic H5N1 vaccines in humans.

## Introduction

Avian-origin H5N1 influenza, which has caused 860 cases of human infection around the world (2003-September 2017, with 454 deaths, data from World Health Organization), has the potential to cause the next influenza pandemic. Availability of effective vaccines against this zoonotic virus is an essential part of pandemic preparedness. However, H5N1 antigens are poorly immunogenic to humans, which necessitates the use of an adjuvant, a substance that augments the immunogenicity of vaccines, in manufacturing pre-pandemic H5N1 vaccines^1–3^.

Until recently, aluminum salts have been the only adjuvants used in human vaccines licensed in the United States^4^. Unlike newer oil-in-water adjuvants such as MF59 or AS03, aluminum salts are inexpensive and safe and have been successfully used in diphtheria, tetanus, and pertussis vaccines for more than 80 years^4,5^. Aluminum salts are also used in hepatitis A, hepatitis B, and human papillomavirus vaccines^6^. Nevertheless, their adjuvanticity for influenza vaccines remains unverified.

In animal models, experiments consistently demonstrate that aluminum salts enhance the immunogenicity of H5N1 influenza vaccines^7^. In contrast, randomized controlled clinical trials of aluminum salts-adjuvanted H5N1 influenza vaccines in humans are either statistically inconclusive – likely due to the insufficient sample sizes in each of these clinical trials – or not specifically designed to evaluate the adjuvanticity of aluminum salts^8^.

To maximize the pandemic preparedness, it is necessary to clarify whether aluminum salts, the approved, safe, and affordable adjuvant, are effective in enhancing the immunogenicity of pre-pandemic influenza vaccines. To overcome the limitation of insufficient statistical power in single randomized controlled trials, we conducted the first meta-analysis on this issue.

## Results

### Literature search

**Figure 1:**
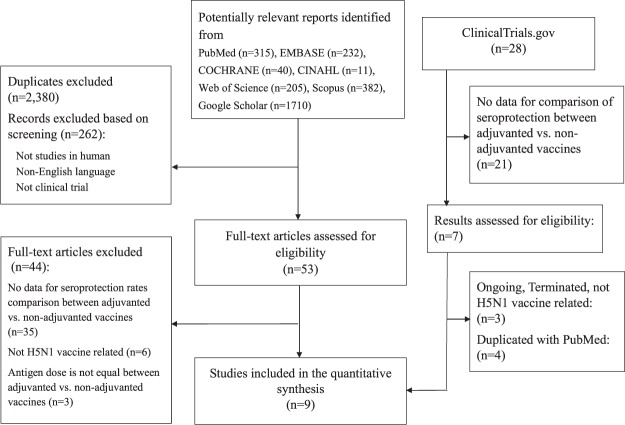
Flowchart of the literature search.

Figure 1 shows the flowchart of the literature search. Trials were eligible if they were randomized controlled trials that compared the immunogenicity of aluminum-adjuvanted H5N1 influenza vaccines versus that of non-adjuvanted counterparts (with the same dose of identical H5 antigen) in healthy individuals. We identified all eligible trials through searching PubMed, EMBASE, Cochrane, CINAHL, Web of Science, Scopus, and Google Scholar, along with reports completed in the clinical trial registry database (ClinicalTrials.gov) before June 30, 2017. We used the following search keywords: “influenza vaccine” AND “aluminum”, filtered by human, clinical trial, and English language. We excluded those trials that did not compare adjuvanted vaccines versus non-adjuvanted counterpart, those that did not involve H5N1 vaccines, and those trials that used different antigen doses across the compared groups.

Of the 2,895 published papers and 28 completed clinical trial reports identified by initial keyword searches, nine non-duplicated randomized trials met the eligibility criteria^1,9–16^. These nine trials (completed during 2006–2013) included 22 comparisons (some trials consisted of several comparisons that assessed different antigen doses of the same vaccines, see Supplementary Table S1 for the details of each comparison), with a total of 2,467 healthy participants. All included trials used a two-dose schedule (with an interval of one month) for testing pre-pandemic H5N1 vaccines. Of the 22 comparisons, 16 and 12 comparisons reported seroprotection rates (proportions of subjects with titers reaching seroprotection levels, see Methods for the definition) by hemagglutination-inhibition assay and by neutralizing titer assay, respectively, 21–28 days after the first-dose vaccination.

### Seroprotection

**Figure 2:**
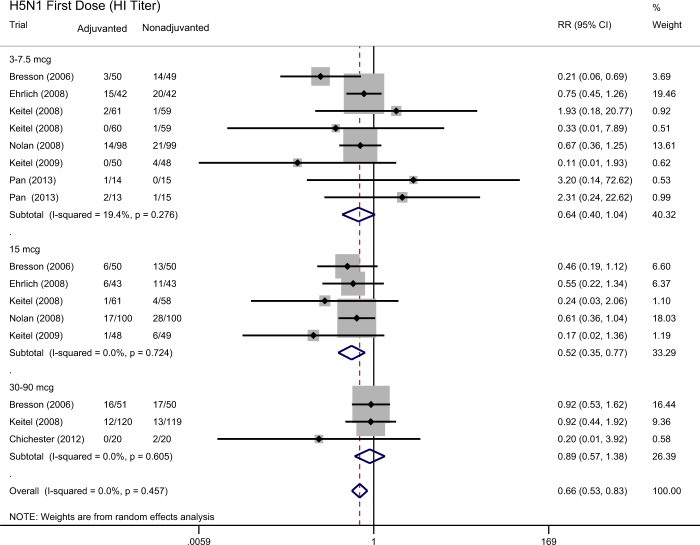
Forest plot showing the ratio of the seroprotection rate by hemagglutinin-inhibition assay, 21–28 days after the first dose of H5N1 vaccines in participants who received aluminum-adjuvanted vaccines versus non-adjuvanted vaccines.

**Figure 3:**
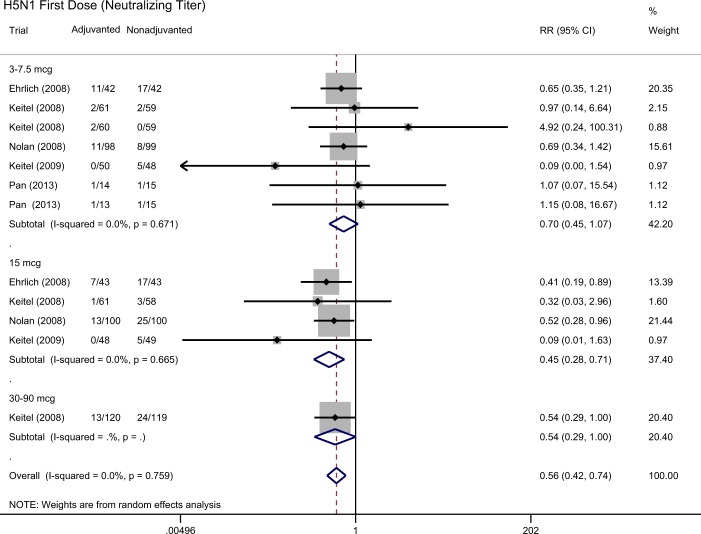
Forest plot showing the ratio of the seroprotection rate by neutralization antibody assay, 21–28 days after the first dose of H5N1 vaccines in participants who received aluminum-adjuvanted vaccines versus non-adjuvanted vaccines.

Compared with non-adjuvanted counterparts, H5N1 vaccines with aluminum salts adjuvant were associated with a significantly lower, rather than higher, seroprotection rate 21–28 days after the first dose. The weighted estimate for the ratio of the seroprotection rate by hemagglutination-inhibition assay was 0.66 (95% confidence interval [CI]: 0.53 to 0.83, I-square: 0.0%) across a range of different antigen doses (Fig. 2). The weighted estimate for the ratio of the seroprotection rate by neutralizing titer assay was 0.56 (95% CI: 0.42 to 0.74, I-square: 0.0%) across a range of different antigen doses (Fig. 3).

After the second dose, aluminum-adjuvanted H5N1 vaccines still did not yield a higher seroprotection rate. The seroprotection rate after the second dose was lower than that conferred by non-adjuvanted counterparts, although the difference was not statistically significant. The weighted estimate for ratios of the seroprotection rate 21–28 days after the second dose of H5N1 vaccine were 0.97 (95% CI: 0.82 to 1.13, I-square: 21.8%) by hemagglutination-inhibition assay (Supplementary Fig. S1) and 0.99 (95% CI: 0.88 to 1.12, I-square: 11.8%) by neutralizing titer assay (Supplementary Fig. S2).

Funnel plot analyses did not show publication biases (Supplementary Fig. S3).

### Harm

**Figure 4:**
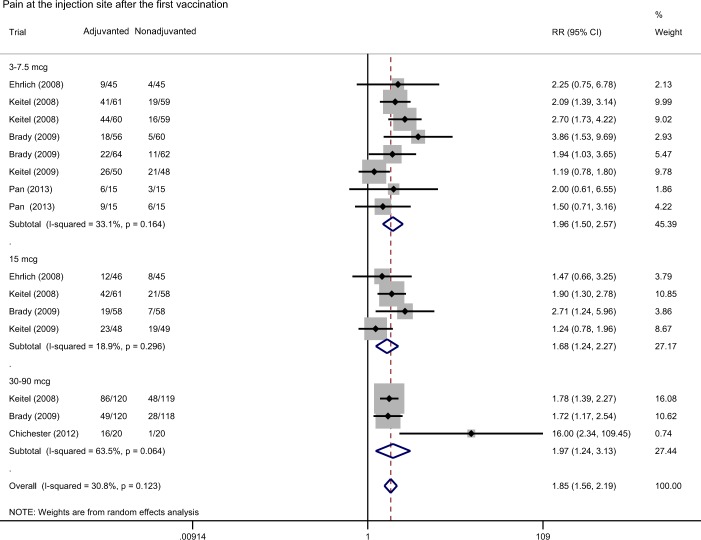
Forest plot showing the risk ratio of pain/tenderness at the injection site during the 7 days after the first dose of H5N1 vaccines in participants who received aluminum-adjuvanted vaccines versus non-adjuvanted vaccines.

Compared with non-adjuvanted counterparts, H5N1 vaccines with aluminum salts adjuvant were associated with a significantly higher risk of pain/tenderness at the injection site during the 7 days after the first vaccination, with the weighted risk ratio of 1.85 (95% CI: 1.56 to 2.19, I-square: 30.8%) (Fig. 4). There was no difference in risk of fever after vaccination (weighted risk ratio 1.00, 95% CI: 0.30 to 3.35, I-square: 0.0%) (Supplementary Fig. S4).

After the second dose, aluminum-adjuvanted H5N1 vaccines were still associated with a significantly higher risk of pain/tenderness at the injection site (weighted risk ratio 1.72, 95% CI: 1.20 to 2.46, I-square: 70.0%) (Supplementary Fig. S5). There was no difference in risk of fever after the second vaccination (weighted risk ratio 0.31, 95% CI: 0.06 to 1.52) (Supplementary Fig. S6).

### Risk of bias assessment

Three trials, Keitel^12^, Ehrlich^10^, and Chichester^15^, had low risk of bias. Bresson^1^ was open-label trial. Pan^16^ was single-blind. Three trials, Bresson^1^, Bernstein^9^, and Brady^13^ did not reported seroprotection data after the first dose vaccination. Two trials, Nolan^11^ and Keitel^14^, did not reported adverse events separately after the first vs. after the second dose. The assessment was summarized in Supplementary Figs S7 and S8.

### Quality of evidence

We used the GRADE approach to assess the quality of evidence, taking risks of bias, risks of random errors, risks of publication bias, and risks of lack of external validity into consideration. We summarized the findings in Supplementary Table S2 (seroprotection) and Supplementary Table S3 (harm). The certainty of evidence was low to very low for seroprotection rate endpoints due to potential outcome reporting bias (only 16 and 12 of the 22 included comparisons reported seroprotection rates data after the first-dose vaccination) and indirectness (seroprotection is a surrogate for real life protection against infection, disease and death) (Table S2), and moderate for local pain/tenderness at the injection sites (Table S3) due to potential outcome reporting bias (only 15 and 5 of the 22 included comparisons reported pain/tenderness and fever after the first-dose vaccination), respectively. The certainty of evidence was very low for fever due to the wide confidence intervals (0.30 to 3.35 and 0.06 to 1.52) as well as potential outcome reporting bias (Table S3).

## Discussion

This is the first meta-analysis of randomized controlled trials on the efficacy of aluminum salts as an adjuvant for pre-pandemic influenza vaccines. Our results showed an inferior seroprotection rate after aluminum-adjuvanted H5N1 vaccines compared with that conferred by non-adjuvanted counterparts. The absence of an increase in seroprotection rates of aluminum salts-adjuvanted vaccines indicates that aluminum salts are not suitable to serve as adjuvants for pre-pandemic H5N1 influenza vaccines for humans.

The observed lack of efficacy might be explained by the Th2 immune response elicited by aluminum salts^5^; for intracellular pathogens, such as novel influenza virus, a Th1 immune response is required instead^17–19^. The significantly worse seroprotection rate observed in the trial participants received aluminum salts-adjuvanted vaccines suggests that aluminum salts actually interfere with the immunogenicity of pre-pandemic influenza vaccines because the wrong type of T cell response is elicited.

The negative impact of aluminum salts on the immunogenicity of pre-pandemic influenza vaccine in human clinical trials is in sharp contrast with the positive animal experiment results in mice and ferret models. Aluminum salts significantly increase the immunogenicity of H5N1 vaccines, measured by both hemagglutination-inhibition and neutralization titer assays, in both mice^20–22^ and ferrets^7,23,24^. This discrepancy between animal experiments and human clinical trials highlights an important limitation of animal models as a testing ground for vaccine development: animal models have different toll-like receptor expression patterns compared with humans^5^. This difference might explain the different effects of aluminum salts in animals and humans. Moreover, there are several well-known differences between species in terms of the pathophysiology and immune responses to influenza virus infection. For example, ferrets are highly susceptible to a wide range of influenza virus isolates, but mice are not. The presence of the *mx1* antiviral gene in mice necessitates the use of specifically adapted influenza virus strains, which could markedly differ from the field virus isolates, in mouse models^25,26^. Each type of animal model has its unique usefulness and limitations in influenza research^27–29^, which makes it impossible to directly generalize animal study results to humans.

With the aim to directly evaluate the effect of adjuvant on immunogenicity, we did not include comparisons of influenza vaccines with different antigen doses in this meta-analysis. Nevertheless, even if multiple-arms comparisons were taken into consideration, the conclusion on the lack of adjuvanticity of aluminum salts for influenza vaccines would be unlikely to change, as shown by a 2009 network meta-analysis study that primarily aimed to identify the best formulation of H5N1 vaccine^30^. This multiple treatment meta-analysis shows that, unlike non-aluminum adjuvants such as MF59 and AS03, aluminum salts did not significantly enhance the immunogenicity compared with non-adjuvanted vaccines (for comparsons using less than 7.5 mcg H5 antigen the risk differences were 0.01 [95% CI: −0.03 to 0.29] by hemagglutination-inhibition and 0.04 [95% CI: −0.11 to 0.34] by neutralizing titer; for comparisons using 15 mcg H5 antigen dose the risk ratio was 1.05 [95% CI: 0.81 to 1.36]^30^. Another meta-analysis^31^ compared the immunogenicity and safety of H5N1 vaccines with different antigen doses but did not present quantitative analysis results on the adjuvanticity of aluminum salts. This 2016 meta-analysis^31^ included only 8 of the 9 randomized trials enrolled in the present meta-analysis, without the trial (with a total of 545 subjects) reported in 2009 by Brady *et al*.^13^, which we included in our meta-analysis.

Currently, eight manufacturers (based in China, Russia, Kazakhstan, Japan, and Australia) provide licensed aluminum-adjuvanted H5N1 vaccines^32^. While the World Health Organization Strategic Advisory Group of Experts (SAGE) on Immunization stated that “studies using Al(OH)_3_ in H5 inactivated vaccines have produced variable results that are less than impressive”^33^, these vaccines are perceived as cost-saving alternatives to the expensive MF59- or AS03-adjuvanted pre-pandemic influenza vaccines. However, our meta-analyses show that, if aluminum salts were not added in the first place, these same vaccines could be more immunogenic against targeted influenza virus strains. The negative impact of aluminum salts on the immunogenicity of H5N1 vaccines might explain the unexpected failure of Emerflu (Sanofi) in 2011^34^. Emerflu^TM^ is a split-virion inactivated pre-pandemic H5N1 influenza vaccine with 30 μg of hemagglutinin and 600 μg of Al(OH)_3_ and was withdrawn from applications for licenses after pre-marketing trials showed that the seroprotection was below the established criteria^34^. Our findings that the addition of aluminum salts decreased, rather than increased, the immunogenicity of pre-pandemic influenza vaccines are highly relevant to vaccine manufacturers, which play an important role in pre-pandemic preparedness.

With the newly emerged threat of avian-origin H7N9 influenza from China^35,36^, H7N9 vaccines have become a priority in research and development for pre-pandemic preparedness. Several teams of researchers are currently testing candidate H7N9 vaccines in animal models^37–39^. One laboratory reported a very good adjuvant effect of aluminum salts for H7N9 vaccines in ferrets^37^. These promising animal data should be interpreted cautiously^40^, as illustrated by the negative impact of aluminum salts on the immunogenicity of H5N1 influenza vaccines shown in this meta-analysis.

An important limitation of our study is that only 7 and 5 of the 9 included randomized controlled trials (16 and 12 of the 22 comparisons) reported seroprotection rates data after the first-dose vaccination, the primary outcome of our meta-analysis, by hemagglutination-inhibition assay and by neutralizing titer assay, respectively. We had contacted with the authors of the remaining trials, but was unable to obtain unpublished data. Nevertheless, funnel plot analyses did not detect evidence for publication biases.

Our meta-analysis of all available data reported from randomized controlled trials in human subjects shows that aluminum salts decrease, rather than increase, the immunogenicity of pre-pandemic H5N1 influenza vaccines. Furthermore, aluminum salts increase local reactogenicity, with pain/tenderness at injection sites. Therefore, aluminum salts should not be recommended as adjuvants for these vaccines. This unexpected, but important, finding highlights the limitation of animal models as the testing ground for developing pre-pandemic influenza vaccines for humans.

## Methods

### Ethical Statement

This is a meta-analysis of published randomized controlled trials reports and is exempted from human subject research review.

### Definition of Seroprotection

Seroprotection is defined as a titer of ≥1:40 (or ≥1:32) by hemagglutination-inhibition assay, as pre-specified by the investigators of each trial report; or a titer of ≥1:40 (or ≥1:20) by neutralizing titer assay, as pre-specified by the investigators of each trial report.

### Main Outcome

The main outcome is the ratio of the seroprotection rate 21–28 days after receiving the first dose of aluminum-adjuvanted H5N1 influenza vaccines versus that of non-adjuvanted counterparts (with the same antigen). A single dose of aluminum-adjuvanted H5N1 influenza vaccines is immunogenic and safe^41^ and meets the licensing criteria for interpandemic and pandemic influenza vaccines in the European Union and the United States when the H5 antigen dose is ≥6 mcg^41^.

### Secondary Outcomes

We assessed other potentially important outcomes, including the ratio of the seroprotection rate 21–28 days after receiving the second dose of aluminum-adjuvanted H5N1 influenza vaccines versus that of non-adjuvanted counterparts; and the risk ratios of (a) pain/tenderness at the injection site during the 7 days after the first dose; (b) pain/tenderness at the injection site during the 7 days after the second dose; (c) fever (body temperature higher than 38 °C) during the 7 days after the first dose; and (d) fever (body temperature higher than 38 °C) during the 7 days after the second dose.

### Statistical Software

Forest plots were generated for summarizing ratios of seroprotection rates using random effect models. We used a funnel plot to detect publication bias. STATA 9.0 (Stata, Stata Corp LP, College Station, TX, USA) was used for all statistical analyses.

### Bias of risk assessment

Risk of bias was assessed using the Cochrane risk of bias tools. It consisted of seven specific domains, including: selection bias (random sequence generation and allocation concealment), performance bias (blinding of participants and personnel and other potential threats to validity), detection bias (blinding of outcome assessment and other potential threats to validity), attrition bias (incomplete outcome data) and reporting bias (selective outcome reporting assessed by comparing outcomes reported in the protocol to those reported in the completed RCT whenever possible)^42^.

### Grade of Evidence

We used the five GRADE considerations to assess the quality of evidence, i.e. risk of bias, inconsistency, indirectness, imprecision, and publication bias^42,43^. We employed GRADEpro (https://gradepro.org/) to create summary tables of the findings for each outcome. We justified all decisions to downgrade or upgrade the quality of evidence using footnotes and comments.

### Data availability

All data analyzed in this study are included in the published article.

## Electronic supplementary material

Supplemental material
