# Supplementary material for: Aluminum salts as an adjuvant for pre-pandemic influenza vaccines: a meta-analysis
Source: Sci Rep. 2018 Jul 30;8:11460. doi: 10.1038/s41598-018-29858-w (PMC6065440; doi:10.1038/s41598-018-29858-w)
Supplement: Supplementary file 1 — Supplemental material [file 41598_2018_29858_MOESM1_ESM.pdf]

Supplemental Material

Supplement to:

Aluminum salts as an adjuvant for pre-pandemic influenza vaccines: a meta-analysis

Yu-Ju Lin[1,2], Yun-Jui Shih[1], Chang-Hsun Chen[1], Chi-Tai Fang[2,3,*]

[1] Division of Preparedness and Emerging Infectious Diseases, Centers for Disease Control, Taipei 100, Taiwan

[2] Institute of Epidemiology and Preventive Medicine, College of Public Health, National Taiwan University, Taipei 100, Taiwan

[3] Division of Infectious Diseases, Department of Internal Medicine, National Taiwan University Hospital, Taipei 100, Taiwan

* Corresponding author: fangct@ntu.edu.tw

Table S1. Characteristics of randomized controlled trials that assessed aluminum-adjuvanted H5N1 vaccines versus non-adjuvanted counterparts

Table S2. Summary of findings: certainty of evidence for seroprotection outcomes

Table S3. Summary of findings: certainty of evidence for adverse event outcomes

Figure S1. Forest plot showing the ratio of the seroprotection rate, by haemagglutinin-inhibition antibody response, 21–28 days after the second dose of H5N1 vaccines in participants received aluminum-adjuvanted vaccines versus non-adjuvanted vaccines.

Figure S2. Forest plot showing the ratio of the seroprotection rate, by neutralization antibody response, 21–28 days after the second dose of H5N1 vaccines in participants received aluminum-adjuvanted vaccines versus non-adjuvanted vaccines.

Figure S3. Funnel plot with pseudo 95% confidence limits.

Figure S4. Forest plot showing the risk ratio of pain/tenderness at the injection site during the 7 days after the second dose of H5N1 vaccines in participants received aluminum-adjuvanted vaccines versus non-adjuvanted vaccines.

Figure S5. Forest plot showing the risk ratio of fever during the 7 days after the first dose of H5N1 vaccines in participants received aluminum-adjuvanted vaccines versus non-adjuvanted vaccines.

Figure S6. Forest plot showing the risk ratio of fever during the 7 days after the second dose of H5N1 vaccines in participants received aluminum-adjuvanted vaccines versus non-adjuvanted vaccines.

Figure S7. Risk of bias summary.

Figure S8. Risk of bias graph.

Table S1. Characteristics of randomized controlled trials that assessed aluminum-adjuvanted H5N1 vaccines versus non-adjuvanted counterparts

**Trial**	**Country**	**Study design**	**Vaccine strain**	**Antigen Dose (mcg)**	**Numbers of participants**	**Age (year)**	**Follow-up time (days)**
Bresson (2006) [1]	France	open-label	A/Vietnam/1194/2004 (H5N1)	7.5	50/49	18-40	42
				15	50/50		
				30	51/50		
Bernstein (2008) [2]	U.S.A.	double-blinded	A/Vietnam/1203/2004 (H5N1)	15	28/29	18-64	56
				30	58/60		
Ehrlich (2008) [3]	Australia, Singapore	double-blinded	A/Vietnam/1203/2004 (H5N1)	7.5	39/42	18-45	42
				15	41/41		
Keitel (2008) [4]	U.S.A.	double-blinded	A/Vietnam/1203/2004 (H5N1)	3.75	61/59	18-49	42
				7.5	60/59		

Trial	Country	Study design	Vaccine strain	Antigen Dose (mcg)	Numbers of participants	Age (year)	Follow-up time (days)
				15	61/58		
				45	120/119		
Nolan (2008) [5]	Australia	double-blinded	A/Vietnam/1194/2004 (H5N1)	7.5	98/97	18-45	42
				15	100/100		
Brady (2009) [6]	U.S.A.	double-blinded	A/Vietnam/1203/2004 (H5N1)	3.75	51/52	≥65	56
				7.5	58/59		
				15	52/56		
				45	111/106		
Keitel (2009) [7]	U.S.A.	double-blinded	A/Vietnam/1203/2004 (H5N1)	7.5	50/48	18-40	42
				15	48/49		

Trial	Country	Study design	Vaccine strain	Antigen Dose (mcg)	Numbers of participants	Age (year)	Follow-up time (days)
Chichester (2012) [8]	U.S.A.	double-blinded	A/Indonesia/05/2005 (H5N1)	90	20/20	18-49	42
Pan (2013) [9]	Taiwan	single-blinded	A/Vietnam/1194/2004 (H5N1)	3	14/15	20-60	42
				6	13/15		

Table S2. Summary of findings: certainty of evidence for seroprotection outcomes

Aluminum adjuvanted pre-pandemic influenza vaccine compared to non adjuvanted pre-pandemic vaccine for people aged over 18 years

Patient or population: people aged over 18 years
Setting: definition of seroprotection
Intervention: aluminum adjuvanted pre-pandemic influenza vaccine
Comparison: non adjuvanted pre-pandemic vaccine

Outcomes	**Anticipated absolute effects*** (95% CI)		Relative effect (95% CI)	№ of participants (studies)	Certainty of the evidence (GRADE)	Comments
	**Risk with non adjuvanted pre-pandemic vaccine**	**Risk with aluminum adjuvanted pre-pandemic influenza vaccine**				
Seroprotection assessed with: HI titer after the first dose follow up: range 21 days to 28 days	178 per 1,000	**118 per 1,000** (94 to 148)	**RR 0.66** (0.53 to 0.83)	1756 (7 RCTs)	⨁⨁◯◯ LOW [a,b]	
Seroprotection assessed with: HI titer after the second dose follow up: range 21 days to 28 days	261 per 1,000	**253 per 1,000** (214 to 295)	**RR 0.97** (0.82 to 1.13)	2467 (9 RCTs)	⨁⨁◯◯ LOW [a,c]	
Seroprotection assessed with: Neutralizing titer after the first dose follow up: range 21 days to 28 days	154 per 1,000	**86 per 1,000** (65 to 114)	**RR 0.56** (0.42 to 0.74)	1716 (5 RCTs)	⨁◯◯◯ VERY LOW [a,d,e]	
Seroprotection assessed with: Neutralizing titer after the second dose follow up: range 21 days to 28 days	331 per 1,000	**327 per 1,000** (291 to 370)	**RR 0.99** (0.88 to 1.12)	2427 (7 RCTs)	⨁◯◯◯ VERY LOW [a,f,g]	

***The risk in the intervention group** (and its 95% confidence interval) is based on the assumed risk in the comparison group and the **relative effect** of the intervention (and its 95% CI).

CI: Confidence interval; **RR:** Risk ratio

GRADE Working Group grades of evidence
High certainty: We are very confident that the true effect lies close to that of the estimate of the effect
Moderate certainty: We are moderately confident in the effect estimate: The true effect is likely to be close to the estimate of the effect, but there is a possibility that it is substantially different
Low certainty: Our confidence in the effect estimate is limited: The true effect may be substantially different from the estimate of the effect
Very low certainty: We have very little confidence in the effect estimate: The true effect is likely to be substantially different from the estimate of effect

Explanations

a. measurement of immunogenicity is a surrogate for real life protection against infection, disease and death
b. Only 16 of the 22 included comparisons reported seroprotection rate data after the first-dose vaccination. We had contacted authors of the 6 comparisons which did not report these data, but were unable to obtain response.
c. Confidence interval 0.82 to 1.13
d. Potential outcome reporting bias
e. Only 12 of the 22 included comparisons reported seroprotection rate data after the first-dose vaccination. We had contacted authors of the 10 comparisons which did not report these data, but were unable to obtain response
f. Confidence interval 0.88 to 1.12
g. Only 18 of the 22 included comparisons reported seroprotection rate data after the second-dose vaccination. We had contacted authors of the 4 comparisons which did not report these data, but were unable to obtain response

Table S3. Summary of findings: certainty of evidence for adverse event outcomes

Aluminum adjuvanted pre-pandemic influenza vaccine compared to non adjuvanted pre-pandemic influenza vaccine for people aged over 18 years

Patient or population: people aged over 18 years
Setting: occurence proportion of pain/tenderness and fever during 7 days after first or second dose
Intervention: aluminum adjuvanted pre-pandemic influenza vaccine
Comparison: non adjuvanted pre-pandemic influenza vaccine

Outcomes	Anticipated absolute effects* (95% CI)		Relative effect (95% CI)	№ of participants (studies)	Certainty of the evidence (GRADE)	Comments
	**Risk with non adjuvanted pre-pandemic influenza vaccine**	**Risk with aluminum adjuvanted pre-pandemic influenza vaccine**				
Pain at the injection site after the first dose assessed with: Yes or No	261 per 1,000	**484 per 1,000** (408 to 573)	**RR 1.85** (1.56 to 2.19)	1669 (6 RCTs)	⨁⨁⨁◯ MODERATE [a]	
Pain at the injection site after the second dose assessed with: Yes or NO	230 per 1,000	**396 per 1,000** (277 to 567)	**RR 1.72** (1.20 to 2.46)	1410 (5 RCTs)	⨁⨁⨁◯ MODERATE [b]	
Fever after the first dose assessed with: Body temperature higher than 38 Celsius degree	35 per 1,000	**35 per 1,000** (11 to 119)	**RR 1.00** (0.30 to 3.35)	282 (3 RCTs)	⨁◯◯◯ VERY LOW [c,d,e]	
Fever after the second dose assessed with: Body temperatyre higher than 38 Celsius degree	52 per 1,000	**16 per 1,000** (3 to 79)	**RR 0.31** (0.06 to 1.52)	228 (2 RCTs)	⨁◯◯◯ VERY LOW [c,d,f]	

***The risk in the intervention group** (and its 95% confidence interval) is based on the assumed risk in the comparison group and the **relative effect** of the intervention (and its 95% CI).

CI: Confidence interval; **RR:** Risk ratio

GRADE Working Group grades of evidence
High certainty: We are very confident that the true effect lies close to that of the estimate of the effect
Moderate certainty: We are moderately confident in the effect estimate: The true effect is likely to be close to the estimate of the effect, but there is a possibility that it is substantially different
Low certainty: Our confidence in the effect estimate is limited: The true effect may be substantially different from the estimate of the effect
Very low certainty: We have very little confidence in the effect estimate: The true effect is likely to be substantially different from the estimate of effect

Explanations

a. Only 15 of the 22 included comparisons reported rate data of pain after the first-dose vaccination. We had contacted authors of the 7 comparisons which did not report these data, but were unable to obtain response
b. Only 13 of the 22 included comparisons reported rate data of pain after the second-dose vaccination. We had contacted authors of the 9 comparisons which did not report these data, but were unable to obtain response
c. Potential outcome reporting bias
d. wide confidence interval
e. Only 5 of the 22 included comparisons reported rate data of pain after the first-dose vaccination. We had contacted authors of the 17 comparisons which did not report these data, but were unable to obtain response
f. Only 4 of the 22 included comparisons reported rate data of pain after the second-dose vaccination. We had contacted authors of the 18 comparisons which did not report these data, but were unable to obtain response

Figure S1. Forest plot showing the ratio of the seroprotection rate, by haemagglutinin-inhibition antibody response, 21–28 days after the second dose of H5N1 vaccines in participants received aluminum-adjuvanted vaccines versus non-adjuvant vaccines.

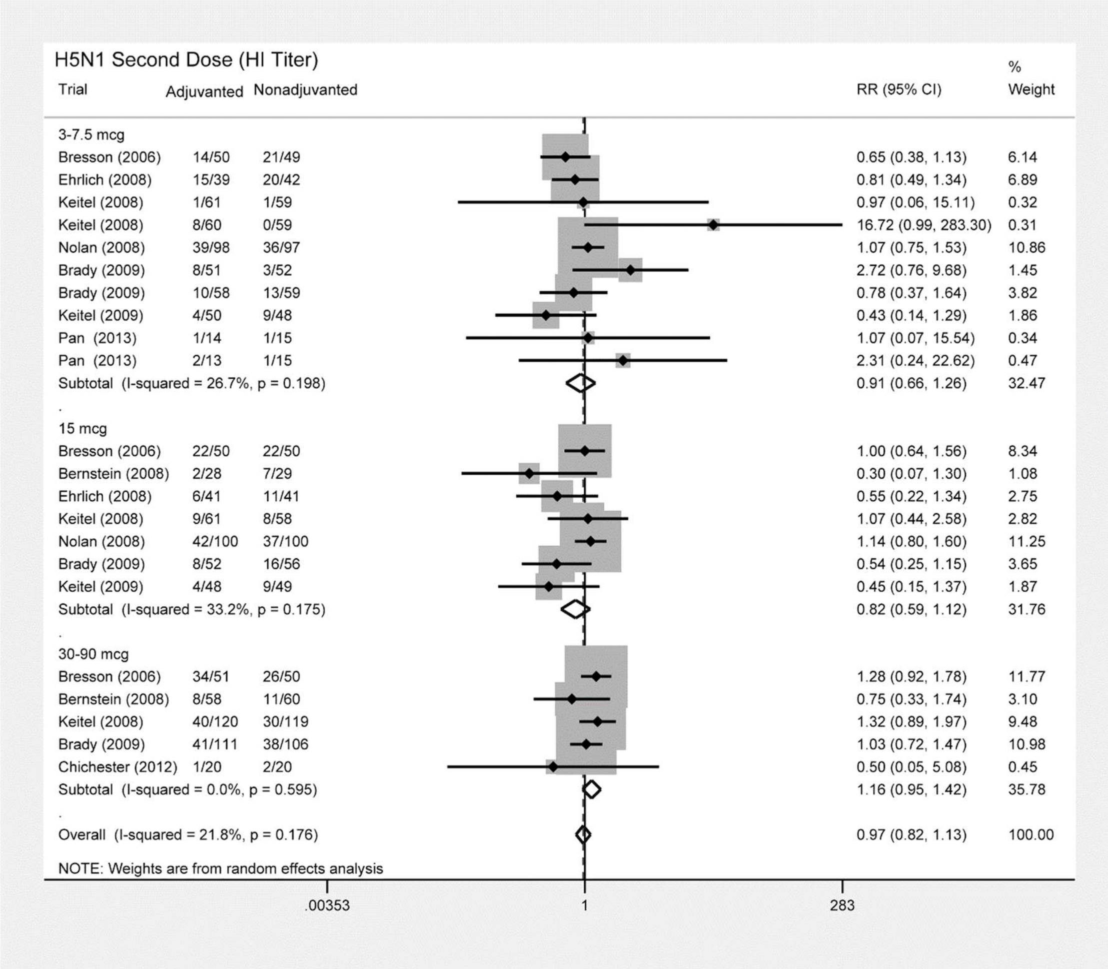

Figure S2. Forest plot showing the ratio of the seroprotection rate, by neutralization antibody titer, 21–28 days after the second dose of H5N1 vaccines in participants received aluminum-adjuvanted vaccines versus non-adjuvant vaccines.

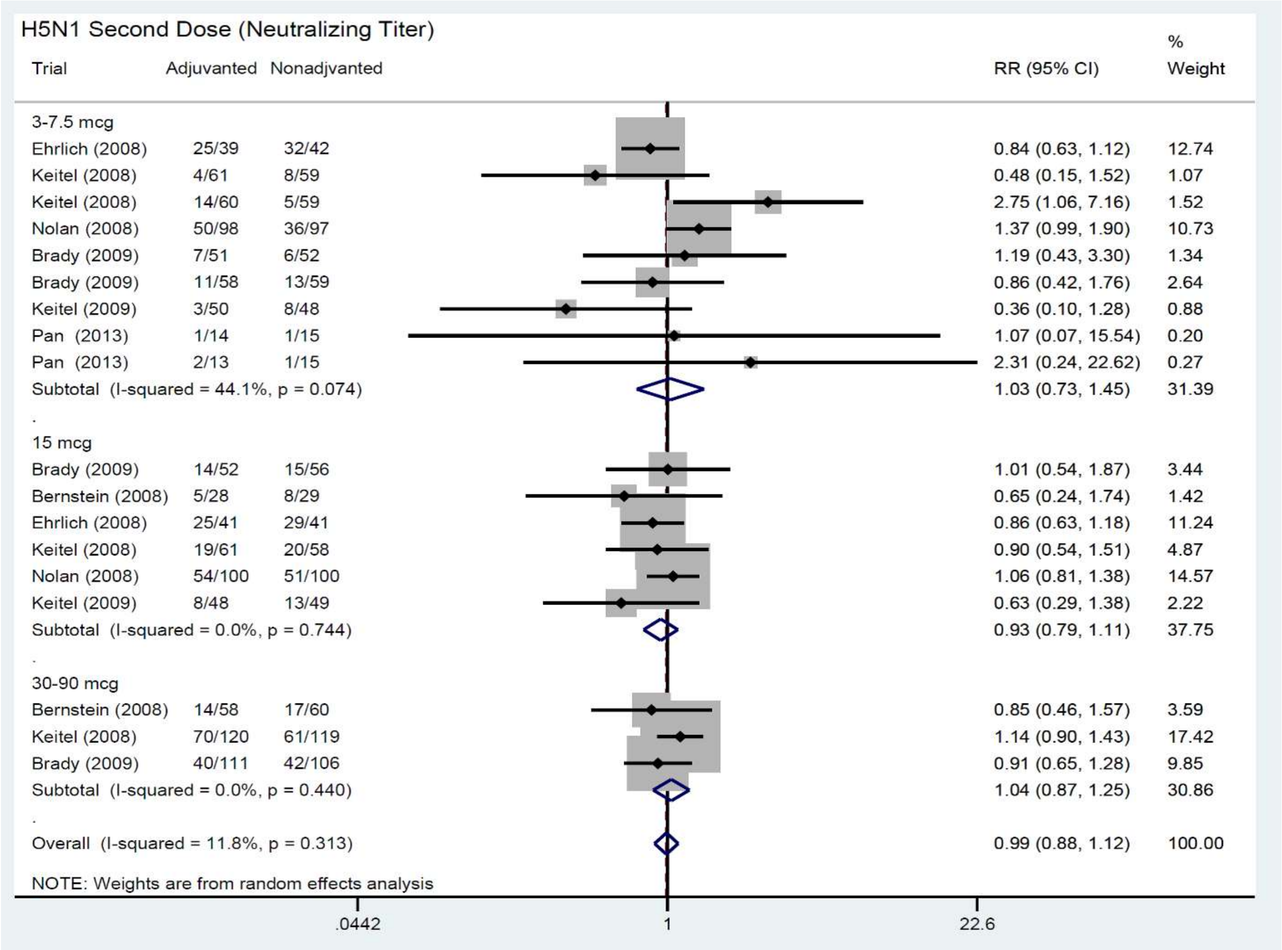

Figure S3. Funnel plot with pseudo 95% confidence limits

(A) Seroprotection rate ratio, by haemagglutinin-inhibition assay, 21–28 days after the first dose of H5N1 vaccines.

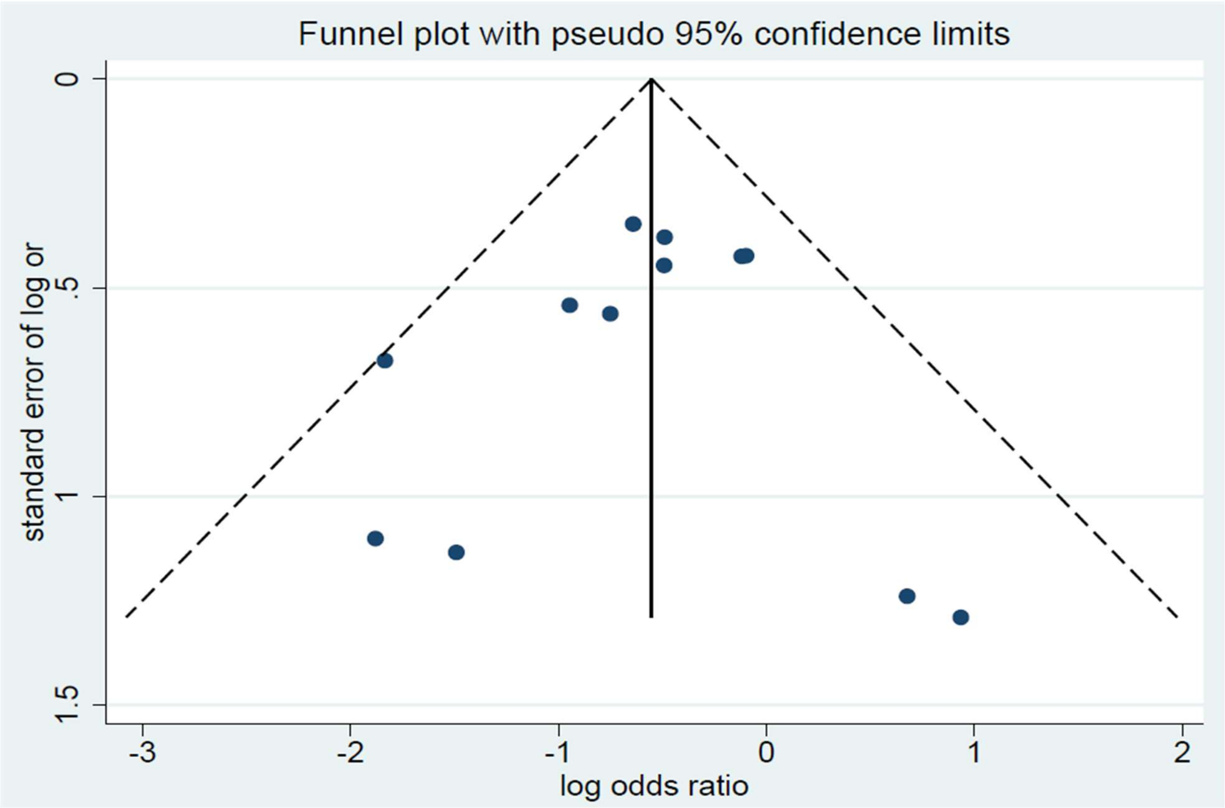

Figure S3. Funnel plot with pseudo 95% confidence limits (Continued)

(B) Seroprotection rate ratio, by neutralizing antibody assay, 21–28 days after the first dose of H5N1 vaccines

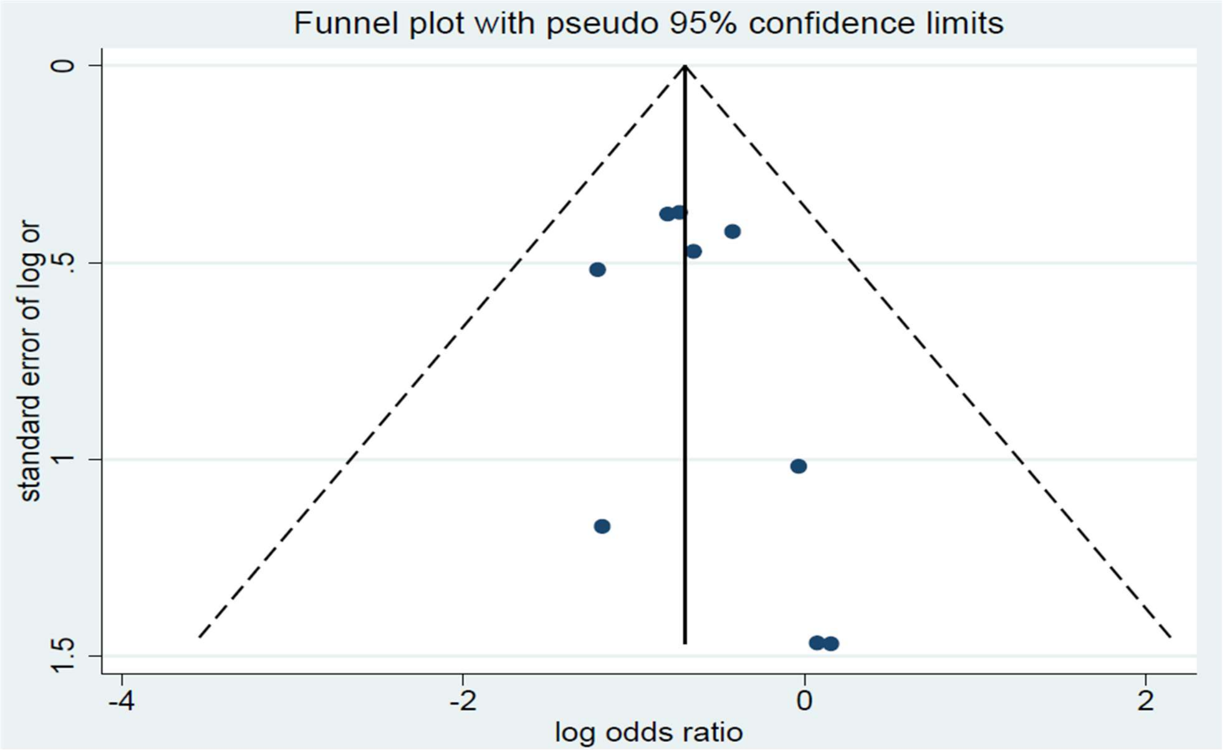

Figure S3. Funnel plot with pseudo 95% confidence limits (Continued)

(C) Seroprotection rate ratio, by haemagglutinin-inhibition assay, 21–28 days after the second dose of H5N1 vaccines

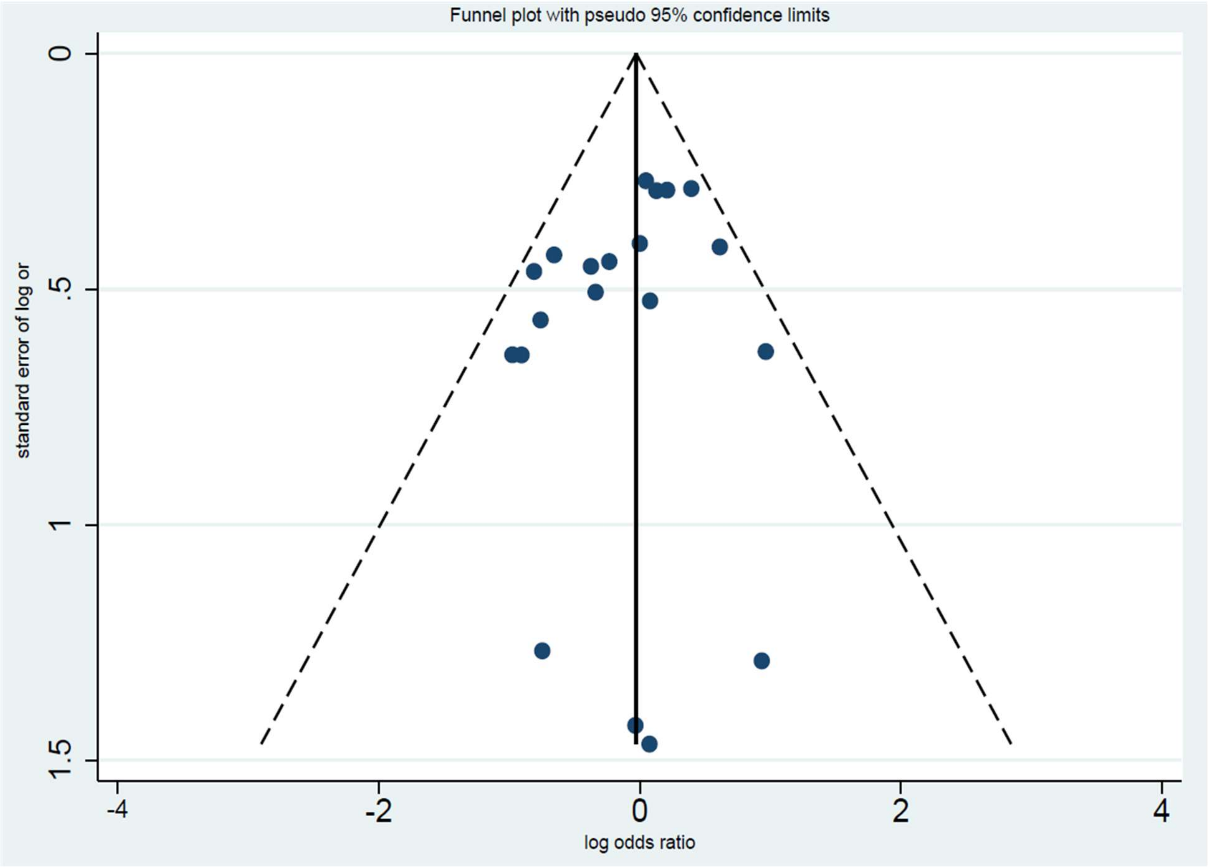

Figure S3. Funnel plot with pseudo 95% confidence limits (Continued)

(D) Seroprotection rate ratio, by neutralizing antibody assay, 21–28 days after the second dose of H5N1 vaccines

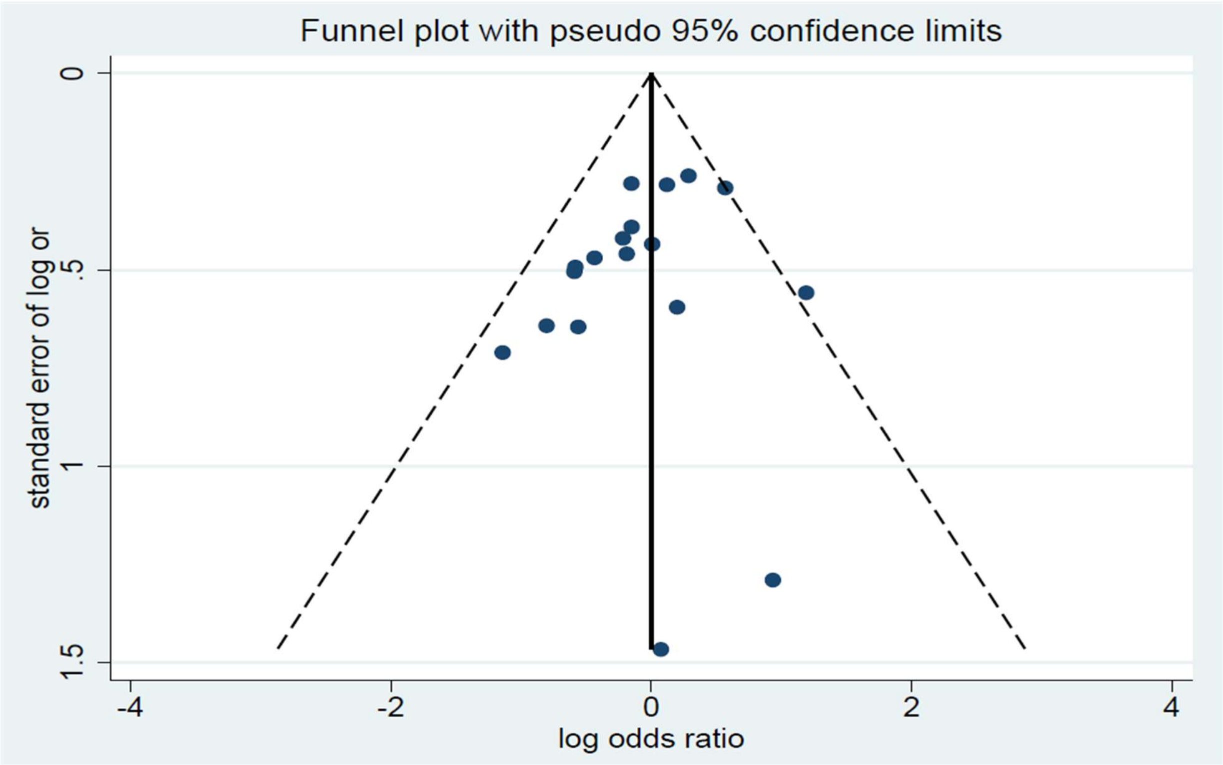

Figure S4 Forest plot showing the risk ratio of pain/tenderness at the injection site during the 7 days after the second dose of H5N1 vaccines in participants received aluminum-adjuvanted vaccines versus non-adjuvant vaccines.

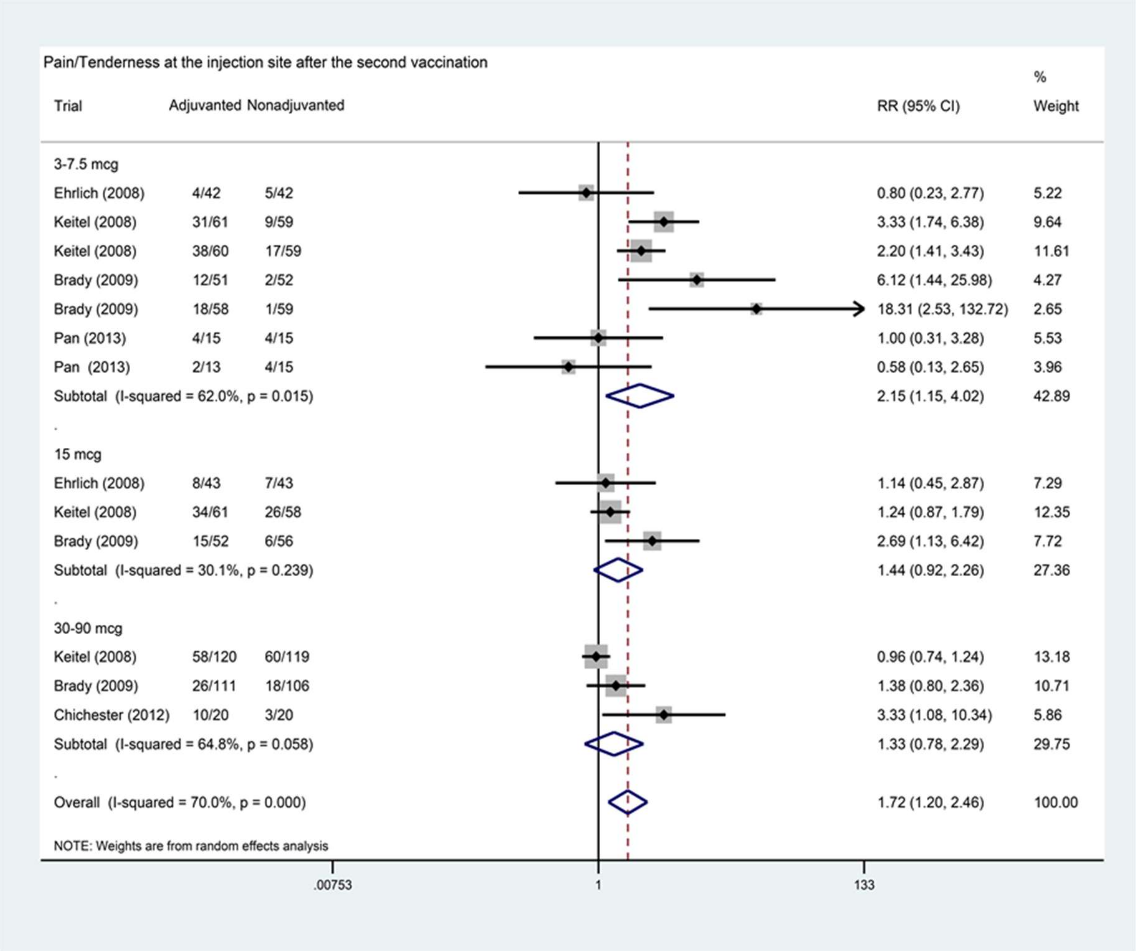

Figure S5. Forest plot showing the risk ratio of fever during the 7 days after the first dose of H5N1 vaccines in participants received aluminum-adjuvanted vaccines versus non-adjuvant vaccines.

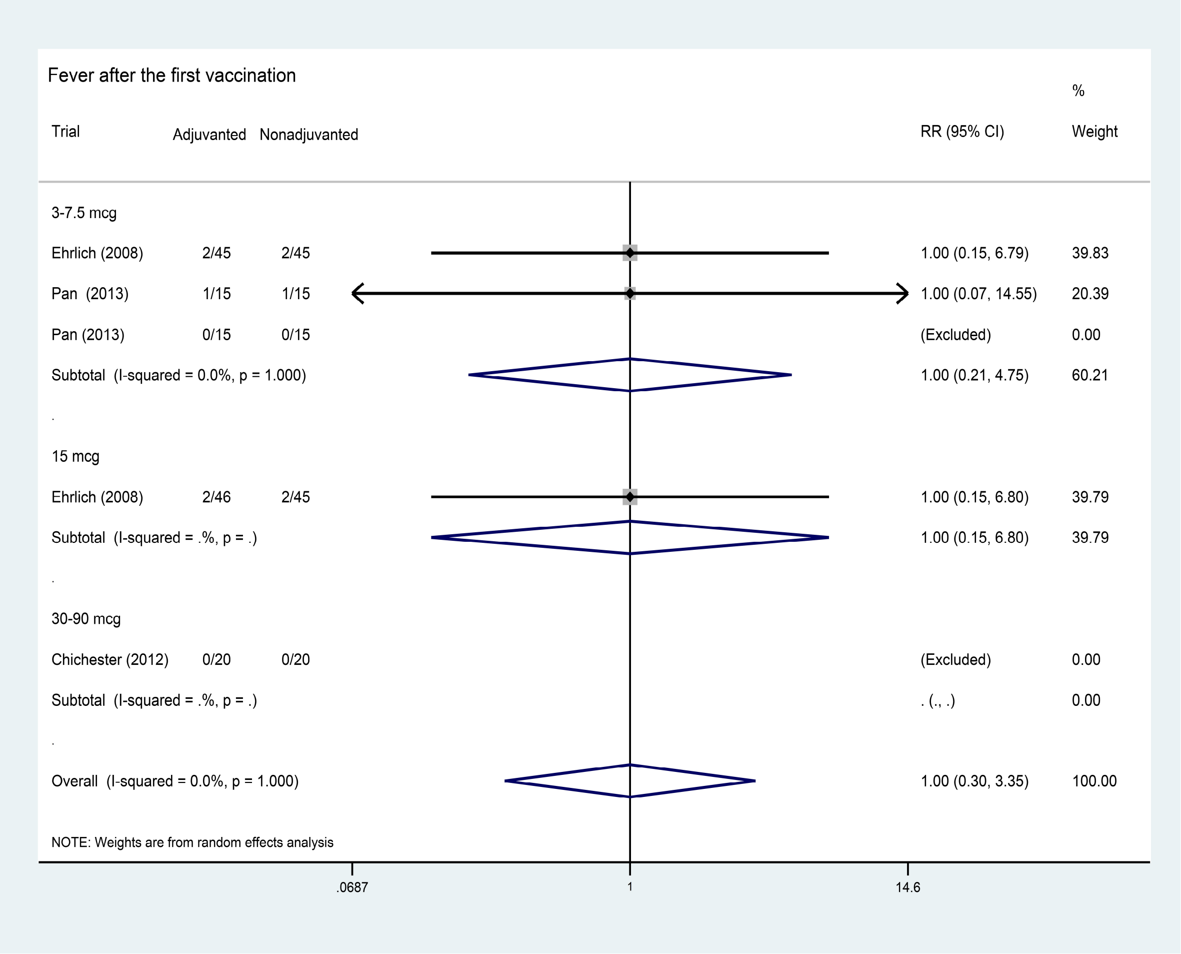

Figure S6. Forest plot showing the risk ratio of fever during the 7 days after the second dose of H5N1 vaccines in participants received aluminum-adjuvanted vaccines versus non-adjuvant vaccines.

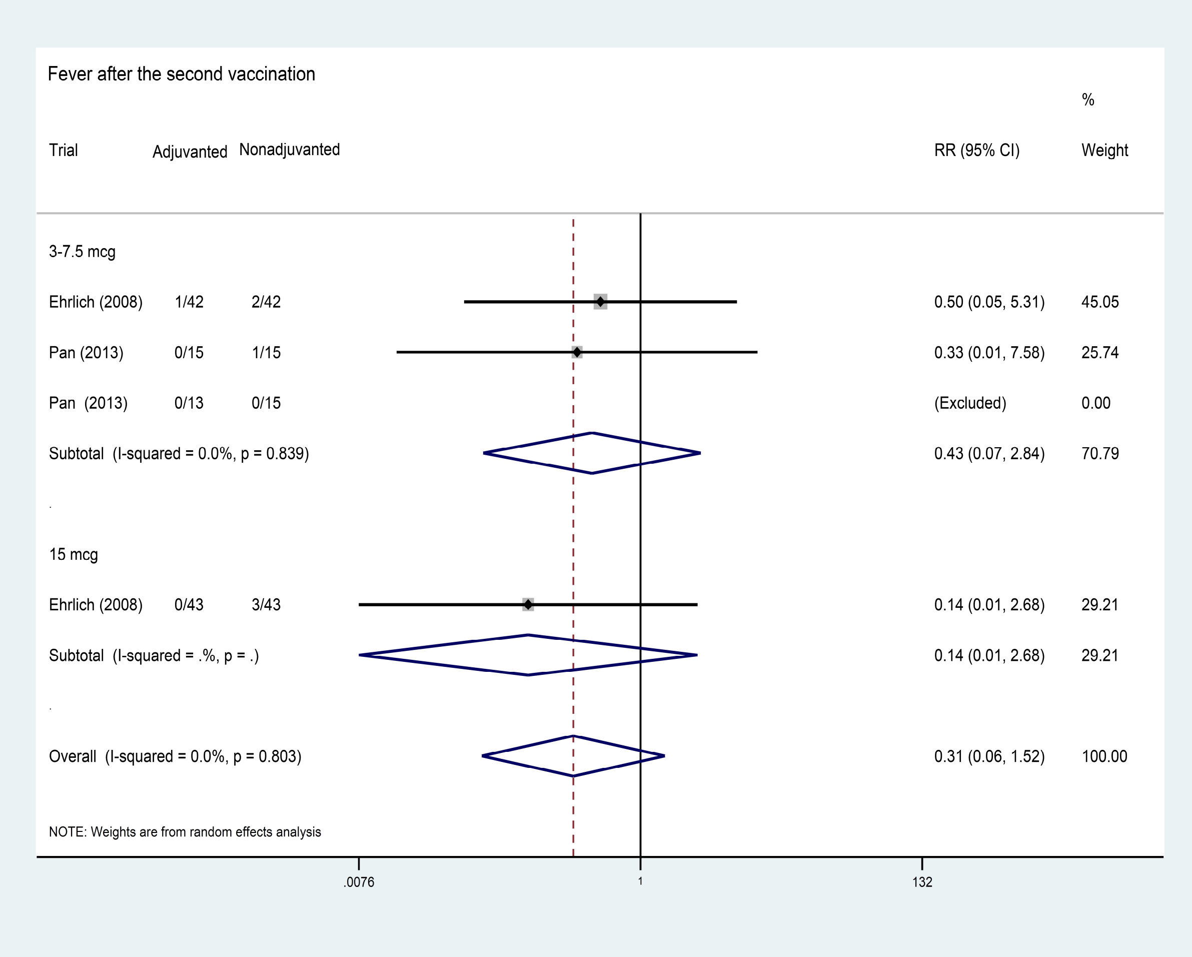

Figure S7. Risk of bias summary.

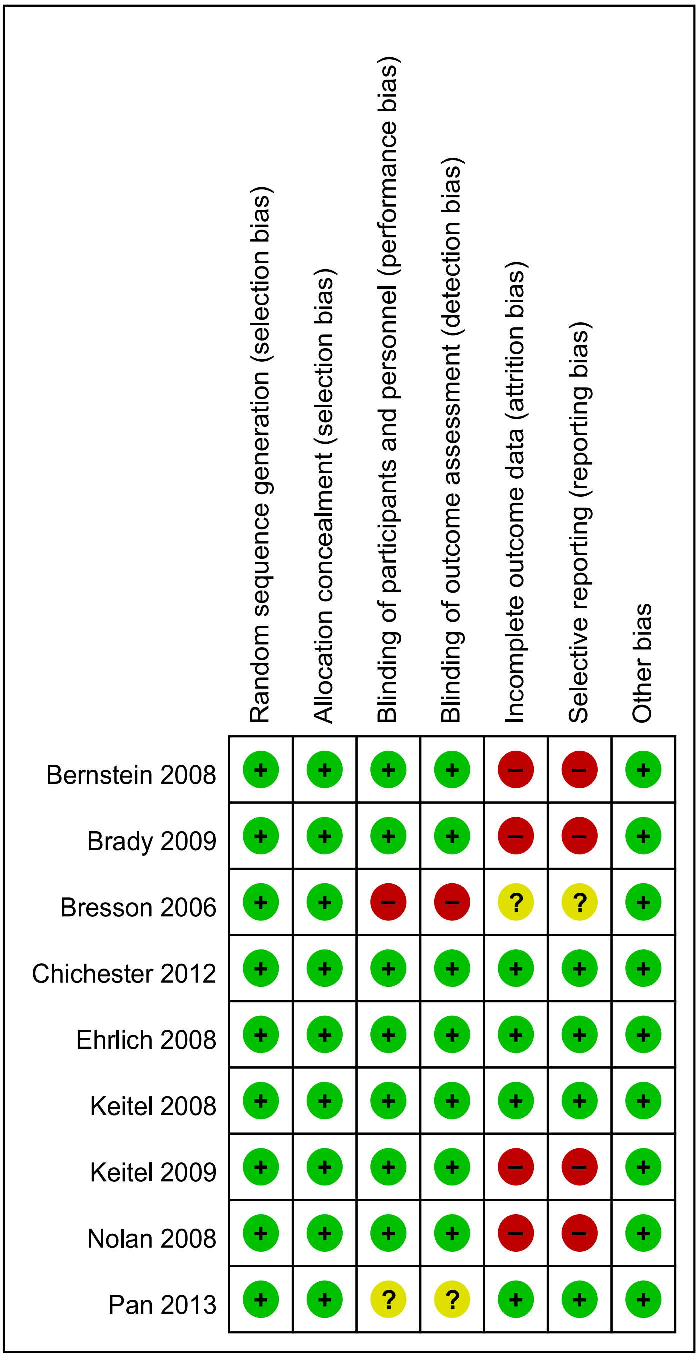

Figure S8. Risk of bias graph.

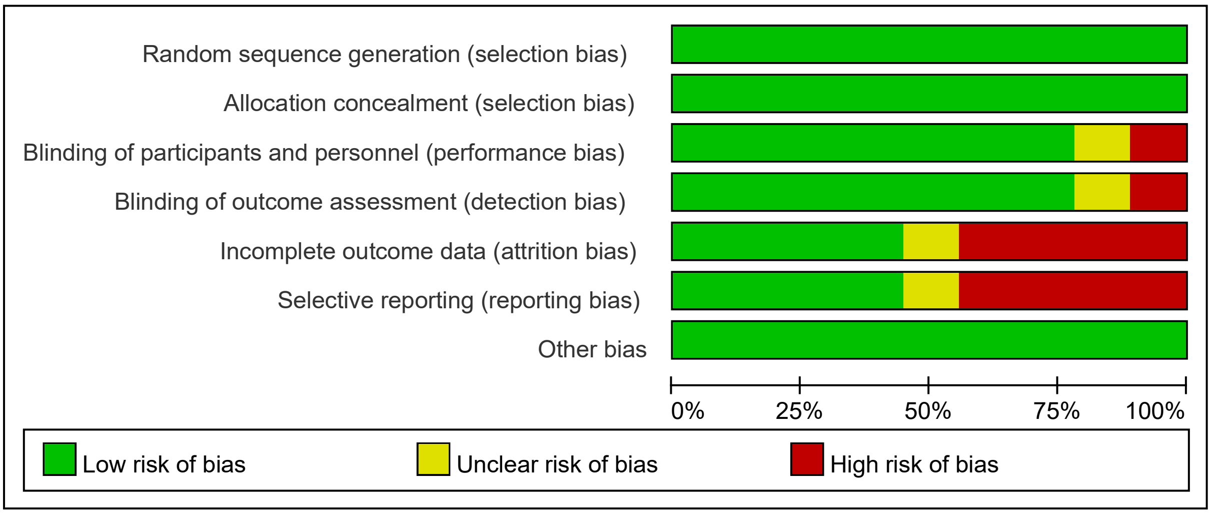

References

1 Bresson, J. L. *et al.* Safety and immunogenicity of an inactivated split-virion influenza A/Vietnam/1194/2004 (H5N1) vaccine: phase I randomised trial. *Lancet* **367**, 1657-1664, https://doi:10.1016/s0140-6736(06)68656-x (2006).

2 Bernstein, D. I. *et al.* Effects of adjuvants on the safety and immunogenicity of an avian influenza H5N1 vaccine in adults. *J Infect Dis* **197**, 667-675, https://doi:10.1086/527489 (2008).

3 Ehrlich, H. J. *et al.* A clinical trial of a whole-virus H5N1 vaccine derived from cell culture. *N Engl J Med* **358**, 2573-2584, https://doi:10.1056/NEJMoa073121 (2008).

4 Keitel, W. A. *et al.* Safety and immunogenicity of an inactivated influenza A/H5N1 vaccine given with or without aluminum hydroxide to healthy adults: results of a phase I-II randomized clinical trial. *J Infect Dis* **198**, 1309-1316, https://doi:10.1086/592172 (2008).

5 Nolan, T. M. *et al.* Phase I and II randomised trials of the safety and immunogenicity of a prototype adjuvanted inactivated split-virus influenza A (H5N1) vaccine in healthy adults. *Vaccine* **26**, 4160-4167, https://doi:10.1016/j.vaccine.2008.05.077 (2008).

6 Brady, R. C. *et al.* Safety and immunogenicity of a subvirion inactivated influenza A/H5N1 vaccine with or without aluminum hydroxide among healthy elderly adults. *Vaccine* **27**, 5091-5095, https://doi:10.1016/j.vaccine.2009.06.057 (2009).

7 Keitel, W. A. *et al.* Safety and immunogenicity of inactivated, Vero cell culture-derived whole virus influenza A/H5N1 vaccine given alone or with aluminum hydroxide adjuvant in healthy adults. *Vaccine* **27**, 6642-6648, https://doi:10.1016/j.vaccine.2009.03.015 (2009).

8 Chichester, J. A. *et al.* Safety and immunogenicity of a plant-produced recombinant hemagglutinin-based influenza vaccine (HAI-05) derived from A/Indonesia/05/2005 (H5N1) influenza virus: a phase 1 randomized, double-blind, placebo-controlled, dose-escalation study in healthy adults. *Viruses* **4**, 3227-3244, https://doi:10.3390/v4113227 (2012).

9 Pan, S. C. *et al.* The Madin-Darby canine kidney cell culture derived influenza A/H5N1 vaccine: a phase I trial in Taiwan. *J Microbiol Immunol Infect* **46**, 448-455, https://doi:10.1016/j.jmii.2012.08.002 (2013).
